# The efficacy and toxicities of intensive induction chemotherapy followed by concurrent chemoradiotherapy in nasopharyngeal carcinoma patients with N_3_ disease

**DOI:** 10.1038/s41598-017-03963-8

**Published:** 2017-06-16

**Authors:** Yingying Zhang, Mingqiu Chen, Cheng Chen, Lin Kong, Jiade J. Lu, Benhua Xu

**Affiliations:** 1 Department of Radiation Oncology, Affiliated Union Hospital of Fujian Medical University, 29 Xinquan Road, Fuzhou, 350001 China; 2 Department of Radiation Oncology, Shanghai Proton and Heavy Ion Center, Fudan University Shanghai Cancer Hospital, 4365 Kangxin Road, Shanghai, 200321 China; 3 Department of Radiation Oncology, Shanghai Proton and Heavy Ion Center, 4365 Kangxin Road, Shanghai, 200321 China

## Abstract

To assess the feasibility, efficacy and safety of 4 cycles of induction chemotherapy (ICT) followed by concurrent chemoradiotherapy (CRT) in nasopharyngeal carcinoma (NPC) patients with N3 disease. ICT consisting of paclitaxel (135 mg/m^2^) and nedaplatin (80 mg/m^2^) given every 3 weeks for 4 cycles followed by cisplatin-based CRT was planned. 22 patients completed 4 cycles of TP regimen ICT and the CRT according to the protocol. After 4 cycles of ICT, the ORR of the primary site was 100% (CR 22.7%, PR 77.3%), and that of the cervical lymph nodes was 95.5% (CR 27.3%, PR 68.2%). After the completion of CRT, the ORR of the primary site was 100% (CR 81.8%, PR 18.2%), and that of the cervical lymph nodes also reached 100% (CR 86.4%, PR 3.6%). The main hematological adverse events were grade 1 to 2 (G1/G2) neutropenia/anemia without febrile neutropenia. The most frequent toxicities during CRT were G1/G2 neutropenia, asthenia, oropharyngeal mucositis and skin injury. The median follow-up time was 46.5 (14 to 75) months. The 3-year PFS, DMFS, LRFS and OS were 81.8%, 81.8%, 100%, and 90.9%, respectively. The results suggest that intensive ICT followed by CRT in NPC patients with N3 disease is effective and well tolerated.

## Introduction

Nasopharyngeal carcinoma (NPC) is sensitive to both radiotherapy (RT) and chemotherapy (CT). The 5-year locoregional control rate reaches ~90% after combined chemoradiation therapy even in patients with locally advanced disease. Despite of such superb long-term locoregional control rate, the risk of distant metastasis (DM) remained at ~40% for locoregionally advanced NPC especially for those with T4 and N3 diseases, even after adding concurrent and neoadjuvant chemotherapy, to RT^1^. For patients with N3 NPC, the incidence of DM reaches 57% and the 3-year distant metastasis-free survival (DMFS) is merely 56%^2^. Adjuvant CT has not been commonly used due to lack of efficacy and high toxicity^3,4^.

Results from the TAX 323 and TAX 324 trials had confirmed that more intense induction chemotherapy (ICT) could significantly improve the treatment outcomes for patients with locally advanced head and neck squamous cell carcinoma (SCC)^5,6^. Although concurrent chemoradiotherapy (CRT) is considered the standard for the management of locoregionally advanced NPC, 2 cycles of ICT followed by a platinum-based CRT is also used in the endemic area^7,8^. Whether such practice could maximize the benefit for patients with N3 NPC who posse the highest risk for distant metastasis is not known. Therefore, we designed this non-randomized prospective phase II study to evaluate the efficacy and tolerability of intensive ICT for NPC patients with N3 disease.

## Materials and Methods

### Patient selection

This trial was approved by the Institutional Review Board of the Fujian Medical University Union Hospital (reference number: 2009KY025). All patients signed an informed consent prior to their enrollment to the trial. All patients were evaluated using magnetic resonance imaging (MRI) with contrast, chest computed tomography (CT), bone scan, and ultrasonography of the abdomen and pelvis prior to the accrual and treatment, and were staged with the 7^th^ edition of the American Joint Committee on Cancer (AJCC) staging manual.

The eligibility criteria included: pathologically diagnosed NPC; stage IVB (T1-4N3M0) based on the AJCC7^th^ staging system; age from 16 to 70 years; Eastern Cooperative Oncology Group (ECOG) status of 0–1; no hematological diseases, white blood cell counts of at least 4 × 10^9^/L, hemoglobin level of at least 80 g/L, platelet counts of at least 100 × 10^9^/L, alanine transaminase (ALT) levels at least 2-fold below the upper limit of normal, and normal kidney function (creatinine clearance rate ≥60 ml/min).

### Induction chemotherapy (ICT)

The study chemotherapy regimen consisted of 4 cycles of ICT consisted of paclitaxel(Jiangsu Yew Pharmaceutical Co. Ltd, Wuxi, China) (135 mg/m^2^ as a 3-hour intravenous infusion) followed by intravenous nedaplatin (Nanjing Xiansheng Pharmaceutical Co. Ltd, Nanjing, China) (80 mg/m^2^ over a period of 0.5 to 3 hours) on the first day of every 3 weeks for all eligible patients. Prophylactic hypersensitivity regimens including 5 mg of dexamethasone and 25 mg of phenergan will be routinely used 30 minutes prior to the paclitaxel infusion.

### Concurrent chemotherapy (CRT)

Concurrent chemotherapy with radiation comprised of single-agent cisplatin dose of 80 mg/m^2^ every 3 weeks. The doses were reduced by 25% in the subsequent course if ≥IV hematotoxicity or ≥III hepatotoxicity, nephrotoxity, or neurotoxity was observed.

### Radiotherapy

All patients had contrast-enhanced CT simulations before and after ICT. Treatment targets and organs at risks (OARs) were defined according to the International Commission on Radiation Units & Measurements (ICRU) Report 62 recommendations. The GTVnx was outlined according to the pre-treatment clinical and radiological findings. The GTVnd was outlined according to the CT/MR results after neoadjuvant chemotherapy. CTV1 encompassed GTVnx with a margin of 0.5–1 cm including the entire nasopharyngeal region and 0.5 cm of the submucosal layer. CTV2 encompassed CTV1 with a margin of 0.5–1 cm including the parapharyngeal and retropharyngeal tissues, the posterior part of the nasal fossae, the posterior wall of the maxillary sinuses, the sphenoid and posterior ethmoid sinuses, the skull base, anterior third of clivus and cervical vertebra, cervical lymph node levels (levels II, III, IV, V)^9^. All plans were generated using a commercial treatment planning system (Eclipse 10.0.1 Varian America). Treatment plans were accepted if 95% of the target volume received the prescribed irradiation dose. The prescribed doses, delivered via IMRT, were 68 Gy to the GTVnx, 66 Gy to the GTVnd, 60 Gy to CTV1, and 54 Gy to CTV2, in 30 fractions.

### Evaluation of response

Weekly physical examination and lab tests were performed during chemotherapy and radiotherapy for all patients. Therapeutic effect evaluations were performed at the beginning of the third cycle of ICT, before the administration of RT, and at 3 months after the completion of CRT. The early effects were assessed according to the response evaluation criteria in solid tumors (RECIST) criteria^10^. The primary site and cervical lymph nodes were evaluated separately. Adverse events were graded according to the National Cancer Institute Common Terminology Criteria for Adverse Events (NCI-CTCAE) version 3.0^11^. The radiation-induced toxicities were assessed according to the Radiation Therapy Oncology Group (RTOG)^12^.

### Follow-up

All patients were assessed every 3 months for the first 2 years after completion of CRT, every 6 months for the next 3 years, then annually thereafter. Each clinical follow-up assessment included a complete history and physical examination, nasopharyngoscopy, MRI of the head and neck, CT scan of the thorax, ultrasonography of the abdomen, and serum EBV DNA test.

### Statistical analysis

The primary end point was overall survival (OS) rates at 3 years. The secondary endpoints included progression-free survival (PFS), local recurrence free survival (LRFS), distant metastasis free survival (DMFS) rates, as well as the objective response rate (ORR), defined as the proportion of patients achieved either a partial (PR) or a complete response (CR). The OS was defined as the time from the date of the initiation of ICT to the date of death from any cause. PFS was defined as the time from the date of the initiation of treatment to the date of the first progression or determination of a second primary malignancy. The LRFS and DMFS were defined as the time from the date of the initiation of ICT to the date of locoregional relapse or distant metastasis. The Kaplan-Meier method was used to calculate the survival rates.

The historical 3-year OS rate for patients with stage N3 NPC was approximately 50%. We projected a 40% OS improvement from 50% to 90%^13^. Twenty-one patients were required for a Type I error rate of 0.05 (1-sided) with 80% statistical power to detect an increase of 40% in 3-year OS. After adjusting for a 10% dropout or loss to follow-up rate, the trial required a total of 23 patients.

All statistical data were analyzed using the Statistical Package for the Social Sciences version 17.0 (SPSS, Inc, Chicago, IL, USA).

## Results

### Patient characteristics

Between September 2009 and September 2014, a total of 24 patients were enrolled in this study. Two patients were excluded from the trial for the following reasons: one patient completed 4 cycles of ICTs but refused concurrent chemotherapy during radiotherapy, another declined chemoradiotherapy after 4 cycles of ICTs. Therefore, 22 patients were eligible for analysis. The median age of all patients was 45 years (range 16–66 years). The details of the patient characteristics are listed in Table 1.

**Table 1:** Patient and disease characteristics.

Characteristics	Cases	Percent(%)
Sex		
Male	19	86.4
Female	3	13.6
Age (years)		
≥40	16	72.7
<40	6	27.3
Histology (WHO)		
Type 1	0	0
Type 2	7	31.8
Type 3	15	68.2
AJCC T-classification		
T1	7	31.8
T2	2	9.1
T3	4	18.2
T4	9	40.9
ECOG performance status		
0	22	100
1	0	0

### Treatment and Response to Treatment

All patients received 4 cycles of ICT as planned without interruption. Eleven patients (50%) completed concurrent chemotherapy as planned. After 4 cycles of ICT, the ORRs for the primary disease and cervical lymphadenopathy were 100% (CR 22.7%, PR 77.3%) and 95.5% (CR 27.3%, PR 68.2%), respectively. After the completion of CRT, both ORRs reached 100% (Table 2).

**Table 2:** Response to Induction Chemotherapy and Chemoradiotherapy.

Response	Induction chemotherapy				Chemoradiotherapy			
	Nasopharynx		Neck nodes		Nasopharynx		Neck nodes	
	Patient No.	%	Patient No.	%	Patient No.	%	Patient No.	%
CR	5	22.7	6	27.3	18	81.8	19	86.4
PR	17	77.3	15	68.2	4	18.2	3	13.6
SD	0	0	1	4.5	0	0	0	0
PD	0	0	0	0	0	0	0	0

### Acute toxicity

The most commonly observed adverse effect of ICT was hematologic toxicity. The main hematological adverse events were grade 1/2 neutropenia, anemia and thrombocytopenia. Two patients developed grade 3 neutropenia, one each after 2 cycles and 3 cycles of chemotherapy. Two patients developed grade 3 neutropenia after 4 cycles of chemotherapy. Nonhematologic toxicities included G1/G2 nausea, fatigue and hepatotoxicity. Three patients experienced grade 3 nausea and vigor. (Table 3)

**Table 3:** Acute toxicity during induction chemotherapy.

	Grade 0		Grade 1		Grade 2		Grade 3		Grade4	
	n	%	n	%	n	%	n	%	N	%
Neutropenia	3	13.6	10	45.5	5	22.7	4	18.2	0	0
Febrile neutropenia	22	100	0	0	0	0	0	0	0	0
Anemia	9	40.9	10	45.5	3	13.6	0	0	0	0
Thrombocytopenia	18	81.8	3	13.6	1	4.5	0	0	0	0
Nausea/vomiting	10	45.5	4	18.2	5	22.7	3	13.6	0	0
Asthenia	7	31.8	12	54.5	3	13.6	0	0	0	0
Diarrhea	22	100	0	0	0	0	0	0	0	0
Neurotoxicity	22	100	0	0	0	0	0	0	0	0
Hepatotoxicity	8	36.4	11	50	3	13.6	0	0	0	0

The most frequent toxicities during CRT were G1/G2 oropharyngeal mucositis, skin injury, asthenia, and neutropenia. Grade 3 mucositis and dermitis were observed in 3 patients. Grade 3 neutropenia occurred in 2 patients. Among them, only one had grade 3 anemia and grade 3 thrombocytopenia with chemotherapy interruption, the other continued with a 25% reduction in the second cycle dose. Grade 4 neutropenia was observed in one patient with chemotherapy interruption and the patient experienced a pulmonary infection. (Table 4).

**Table 4:** Acute adverse events during chemoradiotherapy.

	Grade 0		Grade 1		Grade 2		Grade 3		Grade 4	
	n	%	n	%	n	%	n	%	n	%
Neutropenia	8	36.4	7	31.8	4	18.2	2	9	1	4.5
Febrile neutropenia	22	100	0	0	0	0	0	0	0	0
Anemia	7	31.8	10	45.5	4	18.2	1	4.5	0	0
Thrombocytopenia	15	68.2	1	4.5	4	18.2	2	9	0	0
Nausea/vomiting	7	31.8	9	40.9	5	22.7	1	4.5	0	0
Asthenia	6	27.3	12	54.5	4	18.2	0	0	0	0
Weight loss	1	4.5	18	81.8	3	13.6	0	0	0	0
Diarrhea	22	100	0	0	0	0	0	0	0	0
Mucositis	0	0	7	31.8	12	54.5	3	13.6	0	0
Skin injury	0	0	9	40.9	10	45.5	3	13.6	0	0
Hepatotoxicity	16	72.7	6	27.3	0	0	0	0	0	0

### Follow-up and Survival

The median follow-up time from commencing treatment to the close-out date was 46.5 months (14 to 75 months). The 3-year PFS, DMFS, LRFS, and OS were 81.8%, 81.8%, 100%, and 90.9%, respectively. Five failures were found during follow-up, including 1 patient who experienced relapse in the neck node 42 months after commencing treatment, and was salvaged with surgery, 4 patients who developed distant metastasis alone and were treated by second line chemotherapy. Four patients died, including 3 who died from distant metastasis, 1 patient who died from aspiration pneumonia caused by cranial nerve injury 65 months after commencing treatment.

## Discussion

NPC patients with N3 disease have a poor prognosis due to lower regional control and high incidences of distant metastasis. The possible reason is the presence of micrometastases before treatment. Many researchers suggested chemotherapy with increased intensity to reduce distant metastases to obtain a survival benefit for high-risk NPC patients that present with extensive diseases, such as T4 or N2–3^2^. ICT has been demonstated for its role in the multi-modality management for locoregionally advanced NPC. A meta-analysis from 6 clinical trials of patients with ICT that was conducted by OuYang *et al*. showed that a improved 5-year OS and a reduced the risk of distant metastasis^14^. Recently, a meta-analysis of 19 clinical trials showed ICT improved 5-year PFS and distant control^15^. Xu *et al*. retrospectively collected and reviewed the clinical data of 114 patients with N3 NPC, and demonstrated an improved 5-year PFS with ICT plus CRT compared with the CRT alone arm: ICT + CRT arm, 5-PFS 72%; CRT arm, 54%. The results suggested that ICT plus CRT was more effective than CRT alone for treating N3 disease^16^. However, the optimal number of chemotherapy cycles remains controversial. Lin *et al*.^17^ studied the effect of 2 cycles of ICT with TPF for 40 NPC patients with locally advanced disease. Their results showed a less favorable ORR as compared with the results reported by Bae *et al*. giving three cycles of ICT with TPF (81.6% vs. 97%), which suggested that adding 1 cycle of chemotherapy might further improve the ORR^18^. Paccagnella *et al*.^19^ found similar results in other types of head and neck cancers. While 2–3 cycles of neoadjuvant chemotherapy reduced the risk of distant metastasis in N3 patients, it had not translated into a survival benefit. It is possible that more intensive ICT for the treatment of N3 disease is needed to reduce the incidence of distant failure and improve survival.

The ICT regimen utilized in our study, paclitaxel and nedaplatin (TP), has been shown to be effective and safe in lung cancer, esophageal cancer and other solid tumor patients. Paclitaxel is considered a potent radiosensitizer with a function of inducing cell cycle arrest at the G2/M phase. It has less myelotoxicity than docetaxel. In NPC, paclitaxel demonstrated a response rate of 22%, while the combination with paltinum produces a response rate of 59–76.5%^20^. Nedaplatin, a second-generation platinum agent, has shown excellent efficacy and tolerability. When compared with cisplatin, nedaplatin not only induces less nephrotoxicity and gastrointestinal reactions such as vomiting, but also has more obvious anti-tumor effects. The regimen of nedaplatin plus paclitaxel has verified favorable results with short-term efficacy in patients with metastatic NPC. The PR rate and CR rate were found to be 71.87% and 28.7%, respectively^21^. Recently, an effective schedule of nedaplatin combined with paclitaxel was reported as a concurrent chemotherapy regimen for NPC^22^.

Our neoadjuvant scheme showed good results with regard to tumor response and patient survival. The ORR rates in the nasopharynx (NP) and the neck nodes after 4 cycles of chemotherapy reached 100% (CR 22.7% and PR 77.3%) and 95.5% (CR 27.3% and PR 68.2%), respectively. The 3-year PFS, DMFS, LRFS and OS were 81.8%, 81.8%, 100%, and 90.9%, respectively. These results compare favorably with previous reports on three cycles ICT including two-drug and three-drug scheme^18,23–25^.We postulate 3 reasons for the exciting results. First, Taxanes-based ICT regime is superior to the PF regimen. In a recently published paper, Zhang^26^ found that for patients stage IVB, taxanes-based ICT significantly prolonged the 4-year DMFS by 11.2% (86.1%VS74.9% p = 0.034) and the risk of distant metastasis decreased by above 10%. Second, we used intensive ICT consisting of 4 cycles. Paccagnella^19^ found CR rates increased with each cycle up to cycle 4 (from 19% at the end of the third cycle to 31% at the end of the fourth cycle) and few distant metastases with intensive induction strategies for head and neck squamous cell carcinoma. Third, in our study, 31.8% of the treated patients had stage T1 which perhaps enhance the power of the trial to detect a survival benefit.Similar good results were obtained with TPF regimen as ICT reported by Kong^4^, the subgroup analysis demonstrated the 3-year OS and DMFS were 90.2% and 88%, respectively. The DMFS in our study was somewhat lower than that in the study of Kong^4^. The difference might be associated with the patients characteristics. Stage T4 or N3 patients were analyzed in subgroup compare to only stage N3 patients in our study. Another reason was possible that TPF induction scheme may also contribute to patient survival, espically in patients with N3 stage.

Four cycles of TP ICT were well-tolerated without serious toxicity in our experience, and all enrolled patients completed planned ICT. Grade 3 hematologic toxicity, neutropenia, was only seen in 4 patients (18.2%), although higher than in Kong’s report (18.1%)^4^. However, there was no Grade 4 hematologic toxicity observed during ICT, while grade 3 nonhematologic and gastrointestinal toxicity rates were 13.6%, which was lower than Kong’s study (37.1% and 19.8%, respectively)^4^. During the concurrent treatment phase, the most common toxicities were asthenia, weight loss, mucositis, and skin injury. Our study showed that the incidence of grade 3–4 neutropenia (13.5%), mucositis (13.6%) and skin injury (13.6%) were lower than those reported by Rischin, where the rates were 19% and 31%, 23%, respectively^26^. However, we found that the incidence of grade 2 adverse events including neutropenia, mucositis, and skin injury, at 50%, 86.4, and 86.4%, were higher than those reported by Rischin^26^.

The prevailing use of IMRT in the treatment of NPC has substantially improved the treatment outcome especially in local and regional control. As the effect of concurrent chemotherapy is largely limited to local control, a number of authors have questioned whether the combination of chemotherapy and RT is more favorable as compared to using radiation alone in the era of IMRT and induction chemotherapy. Zhang *et al*.^27^ conclude that it may not be necessary to add concurrent chemotherapy to IMRT after induction chemotherapy. In addition, Sun *et al*.^28^ reported that concurrent chemotherapy with IMRT did not deliver a survival benefit but only increased the adverse effects. Currently no predictive factor could assist in determining the selection of patients who may benefit concurrent chemotherapy with IMRT. Therefore, we consider the addition of concurrent chemotherapy to IMRT remains to be the standard of treatment for locally advanced NPC. However, in the future studies, markers should be developed to predict the necessity and the best utilization of chemotherapy with IMRT. For example, serum Epstein-Barr virus (EBV)-DNA level at diagnosis could be tested for its predictive value for distant metastasis and to be used to guide the use of ICT. And functional imaging could be studied as a marker of the patient’s response to ICT for the potential needs of concurrent chemotherapy. It is also potentially possible that few patients with favorable prognosis could benefit from IMRT alone despite of a locally advanced stage.

## Conclusions

Our results demonstrated both the effectiveness safety profile of 4-cycles of nadaplatin and paclitaxel as ICT for NPC patients with N3 disease. With a median follow-up time of 46.5 months, the 3-year PFS, DMFS, LRRF, and OS rates reached 81.8%, 81.8%, 100%, and 90.9%, respectively. However, our study was limited by the small sample size and probable selection bias.These favorable outcomes warrant further investigation in a randomized trial after longer-term follow-up. Relative randomized controlled clinical trials with large sample size are still needed.
