# Author Correction: The efficacy and toxicities of intensive induction chemotherapy followed by concurrent chemoradiotherapy in nasopharyngeal carcinoma patients with N_3_ disease

**DOI:** 10.1038/s41598-017-17039-0

**Published:** 2017-12-01

**Authors:** Yingying Zhang, Mingqiu Chen, Cheng Chen, Lin Kong, Jiade J. Lu, Benhua Xu

**Affiliations:** 1 Department of Radiation Oncology, Affiliated Union Hospital of Fujian Medical University, 29 Xinquan Road, Fuzhou, 350001 China; 2 Department of Radiation Oncology, Shanghai Proton and Heavy Ion Center, Fudan University Shanghai Cancer Hospital, 4365 Kangxin Road, Shanghai, 200321 China; 3 Department of Radiation Oncology, Shanghai Proton and Heavy Ion Center, 4365 Kangxin Road, Shanghai, 200321 China

*Scientific Reports* **7**:3668; doi:10.1038/s41598-017-03963-8; Article published online 16 June 2017

The Acknowledgements section in this Article was omitted. The Acknowledgements should read: “Supported by grant no. 2014-2-19 from the Youth Innovation Program of Fujian Provincial Health and Family Planning Commission”.

